# Herd immunity drives the epidemic fadeout of avian cholera in Arctic-nesting seabirds

**DOI:** 10.1038/s41598-020-79888-6

**Published:** 2021-01-13

**Authors:** Jacintha G. B. van Dijk, Samuel A. Iverson, H. Grant Gilchrist, N. Jane Harms, Holly L. Hennin, Oliver P. Love, E. Isabel Buttler, Stephanie Lesceu, Jeffrey T. Foster, Mark R. Forbes, Catherine Soos

**Affiliations:** 1 Department of Biology, Carleton University, Ottawa, ON K1S 5B6 Canada; 2 Centre for Ecology and Evolution in Microbial Model Systems, Linnaeus University, 391 82 Kalmar, Sweden; 3 Environment and Climate Change Canada, Canadian Wildlife Service, Gatineau, QC K1A 0H3 Canada; 4 Environment and Climate Change Canada, National Wildlife Research Center, Ottawa, ON K1S 5B6 Canada; 5 Department of Veterinary Pathology, University of Saskatchewan, Saskatoon, SK S7N 5B4 Canada; 6 Environment Yukon, Animal Health Unit, Whitehorse, YT Y1A 4Y9 Canada; 7 Department of Integrative Biology, University of Windsor, Windsor, ON N9B 3P4 Canada; 8 IDvet, 34790 Grabels, France; 9 Pathogen and Microbiome Institute, Northern Arizona University, Flagstaff, AZ 86011 USA; 10 Ecotoxicology and Wildlife Health Division, Environment and Climate Change Canada, Saskatoon, SK S7N 0X4 Canada

**Keywords:** Ecological epidemiology, Diseases

## Abstract

Avian cholera, caused by the bacterium *Pasteurella multocida*, is a common and important infectious disease of wild birds in North America. Between 2005 and 2012, avian cholera caused annual mortality of widely varying magnitudes in Northern common eiders (*Somateria mollissima borealis*) breeding at the largest colony in the Canadian Arctic, Mitivik Island, Nunavut. Although herd immunity, in which a large proportion of the population acquires immunity to the disease, has been suggested to play a role in epidemic fadeout, immunological studies exploring this hypothesis have been missing. We investigated the role of three potential drivers of fadeout of avian cholera in eiders, including immunity, prevalence of infection, and colony size. Each potential driver was examined in relation to the annual real-time reproductive number (R_t_) of *P. multocida*, previously calculated for eiders at Mitivik Island. Each year, colony size was estimated and eiders were closely monitored, and evaluated for infection and serological status. We demonstrate that acquired immunity approximated using antibody titers to *P. multocida* in both sexes was likely a key driver for the epidemic fadeout. This study exemplifies the importance of herd immunity in influencing the dynamics and fadeout of epidemics in a wildlife population.

## Introduction

The termination, or fadeout, of epidemics is expected when the pool of susceptible individuals is depleted via host mortality or the acquisition of lasting immunity^1^. For instance, the 1988 epidemic of phocine distemper virus in harbour seals (*Phoca vitulina*) caused large declines in host density (~ 20,000 seals died), resulting in the fadeout of the outbreak due to lack of susceptible pups entering the population^2^. Measles in humans is a classic example in which severe epidemics are followed by deep troughs in prevalence, leading to local extinction of the virus due to herd immunity^3^. Herd immunity refers to the protection of populations from infection, which is brought about by the presence of immune individuals^4^. In addition to acquired immunity, ecological and/or evolutionary factors may also directly or indirectly play a role in epidemic fadeout^5,6^. For example, the fadeout of the 2010 Rift Valley fever epidemic on cattle farms in South Africa was attributed to a depletion of susceptible hosts after natural infection or vaccination, although the decline in vector-suitable habitat due to a decrease in temperature played an important role in the reduction of disease transmission to susceptible individuals^7^. Increased genetic resistance of the host population is an evolutionary process that may also cause epidemic fadeouts^8^. For example, *Daphnia dentifera* from lakes with recent epidemics caused by the yeast *Metschnikowia bicuspidata* are less susceptible and have lower variance in susceptibility than those from lakes without recent epidemics^9^. Although ecological context influences the epidemic size of *M. bicuspidata*^10^, rapid evolution of host resistance seem to explain the epidemic fadeout in this host-parasite system better than ecological processes, such as a decline in seasonal temperatures^11^. Investigating the processes and factors resulting in epidemic fadeouts in natural populations is important, not only for helping us understand factors precipitating observed disease dynamics, but also for improving our ability to predict when populations may be at increased risk or susceptibility to outbreaks. Such information can be used to develop or evaluate proposed prevention or control strategies where required for conservation purposes.

Avian cholera, caused by the bacterium *Pasteurella multocida*, is one of the most significant infectious diseases affecting wild waterfowl in North America. It has led to mass mortality events in affected populations and species assemblages^12–15^, with single epidemics killing > 50,000 birds^16^. Avian cholera produces acute septicemia in wild birds and poultry^17^, and is spread directly via bird-to-bird contact, environmentally through ingestion or inhalation of aerosolized bacteria in contaminated food and water, and through scavenging of infected carcasses^18^. Depending on the bacterial serotype and strains with these serotypes, infection can lead to mortality within 6–12 h after exposure, although 24–48 h is more common^16^. Epidemics typically occur in wetlands with abundant waterfowl, or at breeding colonies with high bird densities^18^. Although > 190 wild bird species have been reported to be infected by avian cholera, waterfowl are most frequently infected^17^. Over the past half century, epidemics in waterfowl have increased in frequency and geographic distribution in North America^19^. Avian cholera is likely endemic in wild waterfowl in North America, in which the seasonal and geographical patterns of mortality closely follow bird migration patterns^16^. Wild birds are thought to be a source of infection to commercial poultry^20^, with avian cholera causing great economic losses to the poultry industry^21^.

Common eiders (*Somateria mollissima*) are highly gregarious sea ducks that breed in large colonies on marine islands^22^, and are often affected by avian cholera. Since the 1960s, several outbreaks of avian cholera have occurred in breeding common eiders in the USA and Canada^23–25^. Severe annual outbreaks of avian cholera occurred at the largest breeding colony of the Northern common eider (*S. m. borealis*), killing > 6000 nesting females at Mitivik Island (also known as East Bay Island), Nunavut, between 2005 and 2012^26^. Outbreaks killed > 30% of females in 2006 and 2008, and reduced juvenile survival by 90%^27–29^, raising concerns over the potential threat avian cholera might pose to the viability of this eider colony^30,31^.

**Figure 1:**
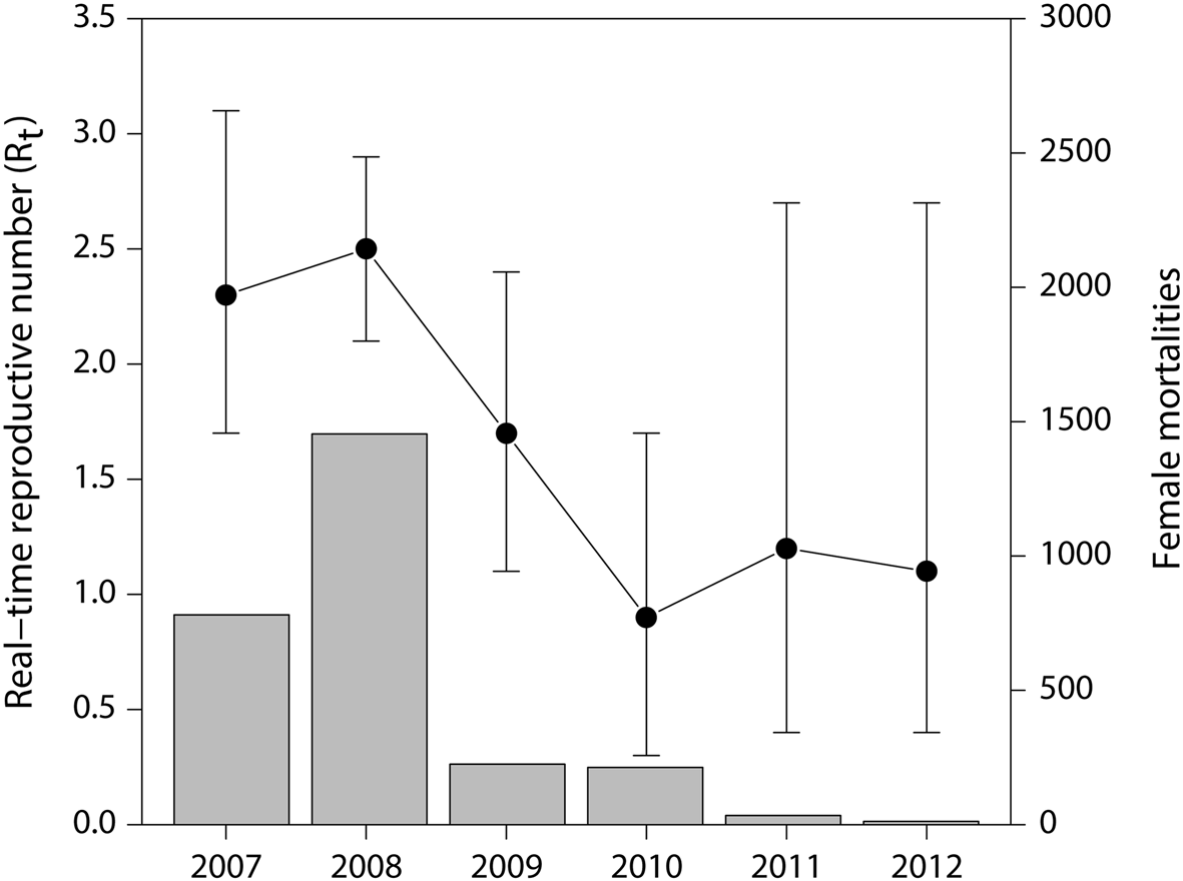
Real-time reproductive number (R_t_) and observed mortalities of the avian cholera epidemic at the Northern common eider breeding colony located at Mitivik Island, Nunavut, from 2007 to 2012. Annual R_t_ (± 95% c.i.) (black dots) calculated by Iverson et al*.*^26^ and the annual number of female mortalities observed (grey bars).

To quantify disease risk to population viability of common eiders at Mitivik Island, Iverson et al*.*^26^ examined the transmission and host population dynamics of the avian cholera epidemic at Mitivik Island between 2006 and 2012. They assessed the basic (*R*_0_) and real-time (*R*_t_) reproductive numbers, which are the expected number of secondary infections generated by each infectious individual in a wholly susceptible population (*R*_0_) or a population ‘in real-time’ when susceptible individuals are depleted by mortality, dispersal and immunity (*R*_t_). They showed an exponential increase in case incidence during the initial wave of exposure, after which the epidemic curve plateaued suggesting a depletion of susceptible hosts, ending with sporadic mortality in the epidemic tail (Fig. 1). Despite a decline of > 50% in eider breeding density, > 4000 breeding pairs persisted after epidemic fadeout, therefore it was suggested that herd immunity played a larger role compared to host population decline in driving epidemic fadeout of avian cholera in this population. The role of host immunity in avian cholera dynamics in Northern common eiders has not been investigated despite its potential to be a major factor in affecting the epidemiology of avian cholera in wild bird populations.

Humoral immunity of hosts plays an integral role in the dynamics of human and livestock diseases^32^, providing information on the exposure history of individuals to infectious agents^33^. Examining host immunity in wildlife, and understanding how host immune response impact disease dynamics are challenging^34^. Avian cholera is an important disease affecting wild bird populations worldwide^35–39^, however few studies have examined host immune response to *P. multocida* infection in wild birds in relation to outbreaks. Samuel et al*.*^40–42^ sampled four species of geese at breeding and wintering sites in Canada and USA showing that seroprevalence or the proportion of geese with antibodies to *P. multocida* serotype 1 ranged between 2 and 8%. Similar seroprevalence of serotype 1 was found in breeding yellow-nosed albatrosses (*Thalassarche carteri*) sampled at Amsterdam Island^43^. Persistence of antibodies to *P. multocida* has been reported to be short-lived based on laboratory infections in ducks^44^, and naturally exposed or vaccinated albatrosses^43^. Studies in captive and wild birds show that vaccination provides some protective immunity against natural exposure to *P. multocida* in unfledged albatross chicks and adult geese^45–47^. Thus, natural infections with *P. multocida* and vaccination both generate adaptive immune responses in wild birds. However, it is unknown whether antibodies mounted in response to natural infections of *P. multocida* confer immunity to subsequent exposures, and if so, whether herd immunity may play a role in explaining the observed dynamics and fadeout of avian cholera outbreaks in the population of Northern common eiders, or other wild bird populations in general.

The overall objective of this study was to evaluate potential drivers associated with the fadeout of annual avian cholera outbreaks in Northern common eiders at Mitivik Island. First, we describe the annual patterns of infection and acquired immunity in apparently healthy Northern common eiders (hereafter called common eiders) sampled upon arrival to Mitivik Island, just prior to the breeding season, and prior to the onset of annual avian cholera outbreaks. Second, we investigate the role of multiple factors that could explain variation in annual R_t_ as calculated by Iverson et al*.*^26^, including measures of acquired immunity (annual seroprevalence and average annual antibody titers) upon arrival, annual proportion of birds with evidence of infection to any strain of *P. multocida* (apparent prevalence of infection), and annual colony size. Given that avian cholera was a novel and highly virulent disease when it was first confirmed in common eiders at Mitivik Island in 2005^31^, and based on the subsequent patterns of annual mortality and eventual disappearance of the disease over time, we hypothesized that herd immunity was the primary driver of the epidemic fadeout of this infectious disease.

## Results

### Patterns of infection and serology

**Figure 2:**
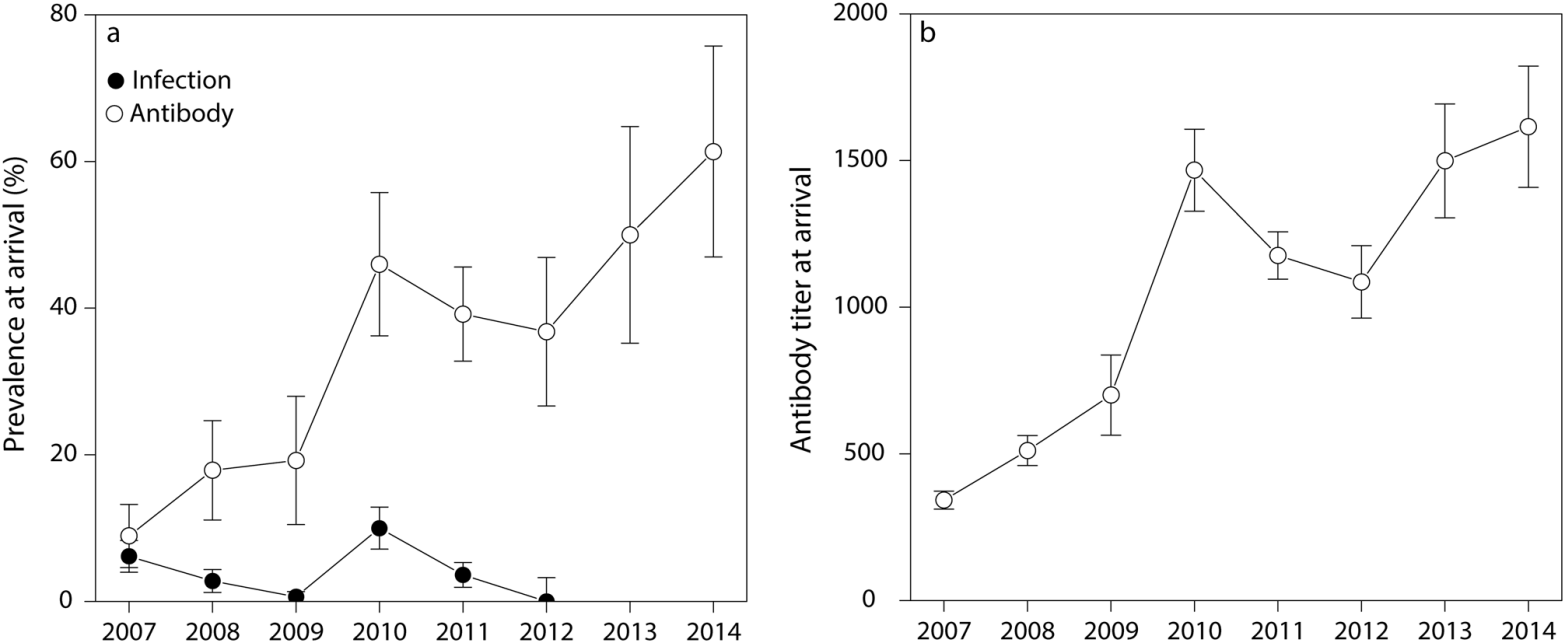
Infection status and serostatus of apparently healthy Northern common eiders upon arrival to Mitivik Island. Annual (**a**) prevalence (± 95% c.i.) of *P. multocida* infection and seroprevalence (± 95% c.i.), and (**b**) average antibody titer (± s.e.m.). N samples infection status: 472 (2007), 430 (2008), 475 (2009), 420 (2010), 469 (2011), 90 (2012). N samples serostatus: 168 (2007), 123 (2008), 78 (2009), 100 (2010), 222 (2011), 87 (2012), 44 (2013), 44 (2014). Note that serostatus of 2013 and 2014 are depicted in the graphs, however these were not included in the models assessing associations with the annual real-time reproductive number (R_t_).

*P. multocida* infection status, assessed by PCR on cloacal and oral swabs, varied annually in apparently healthy common eiders arriving at Mitivik Island from 2007 to 2012 (GLM, X^2^ = 63.07, N = 2356, *P* < 0.001). The highest apparent prevalence of *P. multocida* infection in common eiders was observed in 2010 (10%), whereas almost no birds were infected in 2009 (0.6%) and no birds were detected as infected in 2012 (Fig. 2a). Apparent prevalences in males (3.8 ± 2.7–4.9% (95% c.i.)) and females (4.9 ± 3.7–6.1%) were not statistically different (GLM, X^2^ = 1.59, N = 2356, *P* = 0.207), with no interaction between sex and year (GLM, X^2^ = 5.26, N = 2356, *P* = 0.385).

Serological status and antibody titers, assessed by ELISA, also varied annually in arriving common eiders (GLM, X^2^ = 78.30, N = 778, *P* < 0.001 and LM, F_5,766_ = 45.68, N = 778, *P* < 0.001 respectively). In 2010–2012 a higher proportion of birds had antibodies to *P. multocida,* and antibody titers were higher in those years compared to 2007–2009 (Fig. 2a,b, see Supplementary Figure S1 online for raw data on antibody titers by year and sex). A larger proportion of females (34 ± 30–38% (95% c.i.)) had antibodies compared to males upon arrival (16 ± 12–20%; GLM, X^2^ = 13.99, N = 778, *P* < 0.001). In addition, antibody titers were higher in females (1050 ± 55 (s.e.m.)) compared to males (531 ± 37; LM, F_1,766_ = 11.76, N = 778, *P* < 0.001). This female bias in serological status and antibody titer was true for all years, except 2007 (interaction year and sex: GLM, X^2^ = 11.82, N = 778, *P* = 0.037 and LM, F_5,766_ = 2.79, N = 778, *P* = 0.017 respectively). Serological status (80 ± 45–115%) and antibody titre (1778 ± 394) of common eiders arriving at Mitivik Island in 2006 were excluded from the analysis due to the small sample size (only 5 females sampled in 2006).

### Associations with R_t_

**Figure 3:**
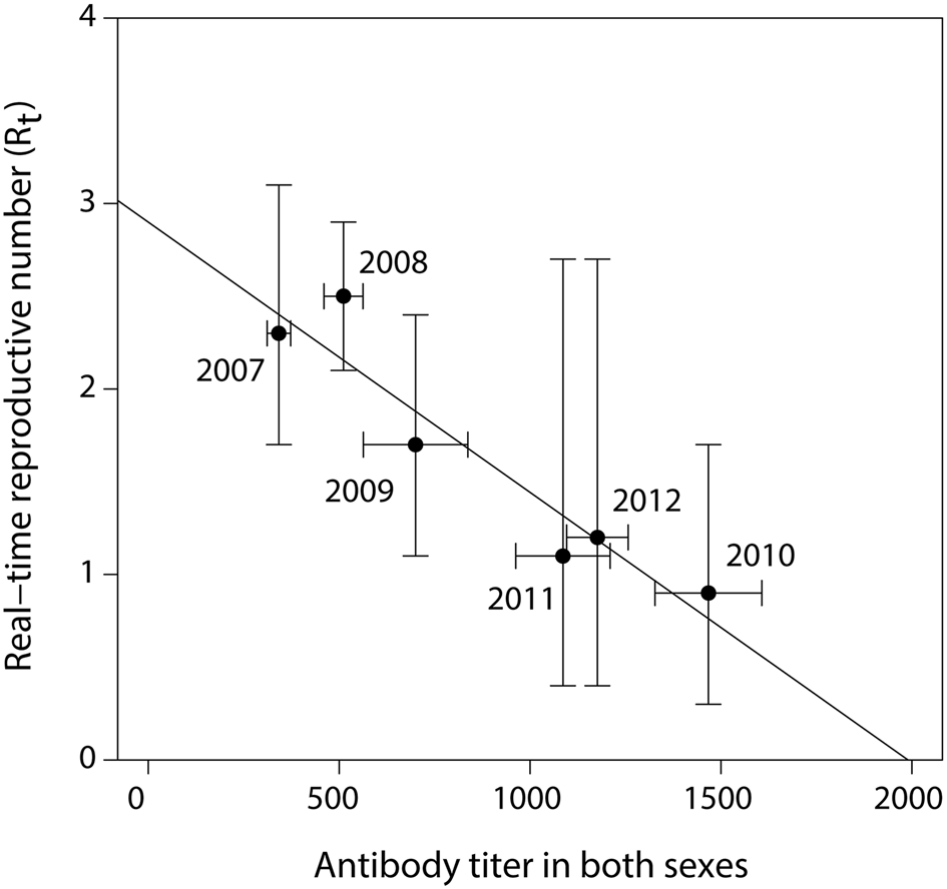
Association between the annual real-time reproductive number (R_t_) (± 95% c.i.) and annual average antibody titer (± s.e.m.) of both sexes of apparently healthy Northern common eiders upon arrival at Mitivik Island, from 2007 to 2012.

The best-ranked model explaining 86% of the variation in R_t_ included the explanatory variable antibody titer measured in both sexes of common eiders (β = − 0.0013 ± 0.0006, N = 6 years, *P* = 0.037). When average annual antibody titers in females and males arriving at Mitivik Island increased, R_t_ decreased (Fig. 3). Lower ranking models included either seroprevalence of both sexes or antibody titer of females, however these models were outranked by the top model by more than 2AIC*c*, and did not improve the null by 2AIC*c* or more, and thus were not viewed as competitive models. Colony size (i.e., number of common eider breeding pairs) and apparent prevalence of *P. multocida* infection did not explain any variation in R_t_ (see Supplementary Table S1 online).

## Discussion

Our study presents results of a rare opportunity to evaluate the theory of epidemic fadeout in a wild population naturally exposed to disease, in the remote Canadian Arctic. Between 2005 and 2012, avian cholera had a devastating effect on the largest breeding colony of common eiders in the Canadian Arctic, reducing eider breeding density at Mitivik Island by more than 50%. Female mortality spiked in 2006, remained elevated in 2007 and 2008, after which it progressively declined to < 1% in 2012^26^. Such patterns generate questions, such as: what caused this disease epidemic to fade out? And can we use this information to design management strategies to prevent such mass mortality events in wild birds in the future? Our study examined potential drivers that could be responsible for this fadeout of annual avian cholera outbreaks. We determined that the likely key driver was the increase in acquired immunity in the common eider population over time. The measure of immunity that explained most of the variation in R_t_ was average annual antibody titers to *P. multocida* in both sexes upon arrival to the island. Other host-specific drivers (i.e., colony size, apparent *P. multocida* infection) were not associated with the fadeout of avian cholera outbreaks on Mitivik Island.

Herd immunity is a phenomenon that slows or halts the spread of a disease in a population, when a sufficient proportion of individuals are immune, resulting in an R_t_ of < 1^4^. Herd immunity likely caused the epidemic fadeout of avian cholera in common eiders at Mitivik Island. We showed that when average annual antibody titers to *P. multocida* increased over the years, R_t_ decreased. Furthermore, following the epidemic fadeout, antibody titers in common eiders remained high in 2013 and 2014 (Fig. 2b). Our results also seem to match the predicted critical proportion of a population that needs to be immune for epidemic fadeout, calculated as 1 − (1/R_t_). The peak value of R_t_ (~ 2.5; Fig. 1) and peak antibody prevalence (~ 60%; Fig. 2a) add up to the predicted threshold value for epidemic fadeout, namely 1-(1/2.5) = 60%. Unfortunately, we could not assess the association between antibody titers and R_t_ for 2013 and 2014, as R_t_ could not be accurately estimated for these years. Although pre-breeding seroprevalence and antibody titers appeared high in 2006, these estimates must be interpreted with caution given that they are based on only 5 females sampled that year. Nonetheless, we can conclude that some eiders had antibodies against *P. multocida* just prior to the onset of the large outbreak observed in 2006 (> 3000 dead). It is unclear whether these were exposed on the colony just prior to the observed outbreak, during the small outbreak observed in the previous year in 2005 (203 dead), or on wintering grounds or stopover sites. According to another study by Iverson et al*.*^48^, it is possible that exposure to *P. multocida* may occur on wintering grounds in Atlantic Canada. Recoveries of common eiders marked with steel leg bands on Mitivik Island and subsequently killed by hunters, as well as movement data for eiders fitted with satellite transmitters on Mitivik Island^49^, indicate that approximately 40% overwinter in Atlantic Canada. The bacterium has been circulating for > 50 years^50^ in common eiders that nest and overwinter in this region. Some of the *P. multocida* serotypes isolated from eiders on Mitivik Island, as well as other parts of eastern Canadian Arctic are the same as those isolated from eiders in Atlantic Canada, however, other serotypes differ among the regions^51^. These results may partially explain the presence of antibody titers in eiders upon arrival to Mitivik Island, however, more detailed research is needed.

*P. multocida* antibodies likely played a role in providing some level of protective immunity to individuals when exposed to the virulent serotypes of avian cholera at Mitivik Island. The main serotypes isolated from female carcasses found at Mitivik Island during the 2007–2012 epidemic varied annually, but were primarily serotype 1, 3 × 4 and 4, with other serotypes (e.g., 3) found less commonly^51^. Although PCR results do not provide information on serotype or virulence, we assume that apparently healthy pre-breeding eiders that tested positive on PCR prior to the onset of the annual outbreak were exposed to serotypes similar to those obtained from carcasses during outbreaks of the same season, or to less pathogenic strains that were cross-protective. Additionally, the serological test employed by our study does not provide information on specific serotypes, but detects antibody responses to any serotype of *P. multocida*. In general, the anamnestic (memory) response after a secondary exposure with the same antigen results in a more rapid production of antibodies, with higher antibody titers^52^. This anamnestic response, either due to multiple exposures to *P. multocida* at the breeding colony at Mitivik Island and/or on wintering grounds in southwest Greenland, or off the coast of Newfoundland or the St. Lawrence estuary may explain the high antibody titers found in both sexes of common eiders upon arrival to Mitivik Island the following year. This would suggest that in contrast to other studies that investigated a single *P. multocida* serotype^43,44^, *P. multocida* antibodies can last longer than a few months, likely due to this anamnestic response resulting from multiple exposures.

Colony size was expected to be an important driver for the epidemic fadeout, because at the end of the avian cholera epidemic in 2012, less than half of the number of breeding pairs was left at Mitivik Island (4570 pairs) in comparison to 2005 (8390 pairs) when the disease was first detected on the island. Nonetheless, during the course of the epidemic years, the proportion of susceptible individuals within the colony decreased, likely because they survived infection and developed protective antibodies to *P. multocida*. This suggests that herd immunity and colony size were operating together to reduce R_t_. Even with a small colony size there could still be locations on the 24-ha island where bird density and hence transmission was high, for instance at higher quality nesting sites near ponds, or locations where eiders aggregate in matrilineal groups. Although the hatching period may conceivably result in an increase in density of susceptible individuals in the colony, mortality of ducklings was rarely detected. The timing and opportunity for exposure of ducklings to *P. multocida* is likely too late in the breeding season to have an effect on the epidemic, as ducklings hatch at the tail end of the outbreaks, and females and ducklings leave the island soon after hatch^53^. Although colony size is seemingly not a key driver for the epidemic fadeout of avian cholera at Mitivik Island, it may well be an important driver for the initiation or propagation of a new avian cholera epidemic in the future. As the proportion of birds with *P. multocida* antibodies decrease over time in concert with increasing population size, and antibody titers decline in the absence of outbreaks, the proportion of susceptible individuals in the population may increase, and its critical density (i.e., epidemic threshold)^54^ may trigger the onset of outbreaks.

Apparent prevalence of *P. multocida* infection in common eiders was not a good predictor for the epidemic fadeout. The main discrepancy between infection prevalence and R_t_ is the year 2010, where in comparison to 2009 infection in common eiders was almost 16-fold higher (10 vs. 0.6%), while R_t_ was almost halved (0.9 vs. 1.7). Although the proportion of infected birds arriving at Mitivik Island increased, avian cholera mortality decreased, likely because almost half of the captured population at arrival had antibodies against *P. multocida* (46%). Hence, high immunity might reflect multiple exposures to less virulent strains. In addition to the increase in immunity over the years, another possible explanation for the reduction in observed mortality over time could also be a decrease in virulence of strains infecting the eider population. Multiple serotypes were responsible for causing mortality in 2007–2009 (1, 3, 4, and 3 × 4)^51^, however only 4 was isolated from female carcasses found in 2010 and 2011. Thus, it is possible that the presence of less virulent strains of *P. multocida* in the population might also have contributed to lower annual mortality as the years progressed.

By including apparent prevalence of *P. multocida* infection in common eiders into our models, we could also investigate whether this variable can be used as an index to examine the annual variation in the initial influx of potentially virulent bacteria into the population at the start of the breeding season. Iverson et al*.*^26^ questioned whether there was a relationship between the proportion of birds carrying a virulent serotype of *P. multocida* (i.e., female mortalities) and PCR-positive birds, which would enable us to use mortality data as surrogate for infection incidence. A potential reason for not finding an association between the two variables is because the PCR test detects any serotype of *P. multocida* (virulent serotypes or not), and the information from female carcasses informs us solely about virulent serotypes. Therefore, a potential effect of the proportion of birds infected by virulent serotypes at the start of the season could have been masked by our test method. Estimates of *P. multocida* infection status are the best data we have for wild populations without experimental controlled exposures, and since we expect inherent variation in our system, we applied a test to evaluate potential drivers associated with the fadeout of annual avian cholera outbreaks.

Our results show a female bias in serological status and antibody titer, but no difference in infection status between the sexes upon arrival to Mitivik Island. The most likely explanation for this difference in acquired immunity is that females are more exposed to *P. multocida* during outbreaks, since they remain on the colony to breed. Males typically leave the island when females start incubating the eggs, prior to or at the beginning of avian cholera outbreaks, and therefore do not experience the same prolonged and repeated exposure to *P. multocida* as nesting females. Observed mortality in males on Mitivik Island was much lower (minimum < 1%) compared to females^26^. Our results also imply that males do not ‘catch up’ in their level of infection to *P. multocida* during the non-breeding season. Since males and females were equally infected upon arrival to Mitivik Island, suggests that both sexes are equally capable of being asymptomatic carriers of *P. multocida*, or were similarly exposed prior to sampling. In addition, the difference in antibody titer between the sexes might also be the result of differences in exposure rate to *P. multocida* during winter if males and females winter in different locations. Information on migration strategies of both sexes and on avian cholera dynamics outside the breeding season are currently too limited^48,49,55^, to allow testing of these hypotheses.

To increase our understanding of the epidemiology of avian cholera in common eiders, a better understanding of eider ecology in the non-breeding season might help us to identify potential sources, or predict future avian cholera outbreaks. Assessing the geographic and seasonal distribution of the genotypes of different *P. multocida* strains, typically grouped by serotype, may assist in this investigation. This information can be helpful in defining strategies to manage and control avian cholera in the future. Although no intervention strategies were employed during the avian cholera epidemic in common eiders at Mitivik Island, future epidemics should be closely monitored, as mitigation or interventions in future outbreaks may be warranted. The eider breeding colony at Mitivik Island was likely large enough to overcome the mass mortalities caused by avian cholera and for the population to build up immunity, but smaller colonies of eiders and other waterfowl are potentially not so robust. An additional threat these bird colonies are currently facing are the frequent incursions of polar bears (*Ursus maritimus*) on islands, which depredate bird nests. Since the 1980s, there has been a sevenfold increase in bear incursions at Mitivik Island^56^, due to the inability of bears to hunt for seals as sea ice coverage in the Canadian Artic is diminishing^57^. Climate-driven changes, such as a change in predation risk and an increase in occurrence of emerging infectious diseases^58^ like avian cholera, will likely affect wildlife populations in the future, posing challenges to conservation biologists to undertake intervention strategies. With this study, we highlight the importance of herd immunity in explaining the dynamics and eventually fadeout of disease epidemics in wildlife populations.

## Methods

### Study species and site

Northern common eiders breed on marine islands throughout the eastern Canadian Arctic and West Greenland. Common eiders only come ashore to nest, whereas in winter they disperse along shallow seashores in flocks of up to several thousands of birds^59^. Nesting phenology is tightly correlated with the timing of sea ice break-up^60^. Females have high fidelity to their natal and breeding areas, and are solely in charge of egg incubation and parental care. Males follow females to their breeding colonies, but leave the colony to molt once females start incubating eggs^22^. After eggs hatch synchronously, ducklings form large amalgamations (crèches) in the water near the breeding colonies^53^. Incursions of polar bears onto eider nesting colonies have greatly increased in frequency over the last decades, with major egg losses from nest depredation^56^.

Mitivik Island (64° 030 N, 81° 789 W) is a 24-ha rocky island, located in the East Bay Migratory Bird Sanctuary, Southampton Island, Nunavut. The island is characterized by low tundra vegetation with shallow freshwater ponds used as drinking water by common eiders throughout the nesting season^61^. In spring, common eiders migrate to Mitivik Island from their wintering grounds in southwest Greenland, in Atlantic Canada off the coast of Newfoundland and within the St. Lawrence estuary^49^. Approximately 4500 eider pairs breed on Mitivik Island annually, making it the largest common eider breeding colony in the Canadian Artic^62^. In addition to common eiders, the island also serves as a breeding ground for smaller numbers of king eiders (*S. spectabilis*), Canada geese (*Branta canadensis*), brant geese (*B. bernicla*), herring gulls (*Larus argentatus*), snow buntings (*Plectrophenax nivalis*), and as migratory stopover in early summer for low numbers of lesser snow geese (*Chen caerulescens*) and Ross’s geese (*C. rossii*)^27^.

### Sampling and counts

Since 1996, Environment and Climate Change Canada has been running a capture-mark-recapture program to monitor the common eider breeding population on Mitivik Island. Each breeding season, biologists are present on the island, before eiders arrive (May) until most of the population leaves (early August). Pre-breeding eiders are captured in flight nets (6 × 100 m) when arriving and flying over the island (mid-June - mid-July), banded, sexed and measured^61^. Across the island, observation blinds are strategically placed to monitor 5 permanent plots (breeding habitat of 15,270 m^2^) to estimate annual number of nest initiations. The location and status of nesting eiders are assessed in each permanent plot by twice-daily scans using binoculars and spotting scopes.

After the discovery of avian cholera on Mitivik Island, samples to assess *P. multocida* exposure were taken from common eiders captured in flight nets upon arrival to the island between mid-June and mid-July, from 2007 to 2014 (N = 2455). Cloacal and oral samples (N = 2356) were collected during the outbreak-period (2007–2012) using sterile polyester-tipped applicators and stored individually in transport medium (sterile Trypticase Soy Broth (TSB) with 15% v/v glycerol). Samples were stored frozen in liquid nitrogen at -196 °C in a cryoshipper in the field. Blood samples were collected from a subset of captured common eiders during the outbreak-period (2007–2012: N = 778) and two years after (2013–2014: N = 88). Blood was taken from either the tarsal vein, or wing or jugular veins and placed into either heparinized vials or vials with no additive, after which it was centrifuged to harvest plasma or serum, respectively, stored frozen at -20˚C in the field. Cloacal, oral and blood samples were stored at − 80 °C until analysis in the lab.

### Bacterial detection

We used a 5′ Taq nuclease PCR assay targeting a portion of the 16S rRNA gene in *P. multocida* to detect the presence of this bacterium in cloacal and oral samples^63^. The 2007–2009 samples were analysed at the Pathogen and Microbiome Institute at Northern Arizona University. The 2010–2012 samples were analysed at the Microbial Ecology and Molecular Diagnostics Laboratory of the University of Saskatchewan, using standardized methods on DNA extraction and amplification described in the Supplementary Methods online. In this study, birds were considered PCR-positive for *P. multocida* when either their cloacal or oral sample was positive on PCR analysis.

### Serology

Serum and plasma samples were transported frozen to IDvet (Grabels, France) where the presence of antibodies directed against *P. multocida* in eider sera and plasma was tested there using an indirect ELISA (ID Screen *Pasteurella multocida* Duck Indirect Test Kit). This ELISA was validated to detect antibody responses to the major *P. multocida* serotypes 1, 3, 4 and 3 × 4. Each plate contained two positive and two negative controls. The absorbance was measured at 450 nm using a Tecan Sunrise plate reader (Tecan, Männedorf, Switzerland). Sample-to-positive ratio (i.e., the absorbance of the samples minus the mean absorbance of the negative control divided by the mean absorbance of the positive minus the negative control; S/P ratio) > 0.5 were considered positive for the presence of antibodies to *P. multocida*. Serostatus, which indicates previous exposure (i.e., positive or negative), was used to estimate the annual seroprevalence. The S/P ratio was used to calculate the antibody titer following manufacturer’s instructions (titer = 10^1.2*log10(S/P ratio)+3.36^), with titer > 997 considered positive. From observations made by one of us (S.L.), *P. multocida* antibody titers do not vary between sera and plasma samples.

### Statistical analysis

The 6-year common eider dataset (2007–2012) was used to evaluate patterns in annual apparent prevalence of *P. multocida* infection, annual seroprevalence and annual average antibody titer (see Supplementary Table S2 online). This dataset was also used to evaluate the role of apparent prevalence of *P. multocida* infection, seroprevalence, antibody titer and colony size in explaining variation in R_t_. Annual colony size was determined using estimates of the total number of nests within the area of permanent plots, extrapolated to the total island area suitable for nesting (19.44 ha).

We used R_t_ estimated by Iverson et al*.*^26^ as a measure of the reproductive number of annual avian cholera outbreaks in common eiders at Mitivik Island from 2007–2012. In short, R_t_ was derived from annual *P. multocida* epidemic curves that were based on female mortalities recorded by biologists from observation blinds (carcasses were counted during twice-daily scans within permanent monitoring plots) (Fig. 1). The estimation of R_t_ from the epidemic curves was done using a maximum-likelihood procedure, which fitted a curve to the mortality data. The curve had three parameters, R_t_, the mean generation time (GT), and the variance in GT. The model used priors for mean GT and variance in GT and then calculated a combination of the three parameters that optimized fit. Sensitivity analyses were conducted to determine the variability of the results for the final R_t_, GT mean, and GT variance depending upon the priors that were used. R_t_ could not be estimated for 2013 and 2014 as mortality data were very sparse (minimum mortality 2013: N = 6, 2014: N = 29). None of the common eider explanatory variables tested here (i.e., apparent prevalence of *P. multocida* infection, seroprevalence, antibody titer, colony size) were part of the calculation of R_t_. Collinearity between explanatory variables was tested with Pearson correlation (*r*; see Supplementary Table S3 online). Although annual seroprevalence and average annual antibody titer were highly correlated (*r* = 0.99), both were examined separately in models to assess associations with R_t_.

To assess the role of year and sex on variation of *P. multocida* infection status and seroprevalence in common eiders, generalized linear models (GLMs) were used (binominal model with logit link function). We included year and sex as fixed factors, and the interaction between year and sex. To assess the variation in antibody titers, we used a linear model (LM), with year and sex as fixed factors, and the interaction between year and sex. Antibody titer was LN-transformed to meet the assumption of normality. Tukey’s post hoc tests were performed to detect differences in infection status, serological status and antibody titer among years.

We used model selection based on the Akaike Information Criterion corrected for small sample sizes (AIC*c*)^64^ to assess the contribution of each of the four explanatory variables (i.e., apparent prevalence of *P. multocida* infection, seroprevalence, antibody titer, colony size) in explaining R_t_. To minimize the chance of over-parameterization (6 data-points: 2007–2012), we only included one explanatory variable in each model. Where possible, analyses were conducted using female data only, male data only and data of both sexes combined. We identified explanatory variables as non-informative when their ΔAIC*c* were > 2 units relative to the null model. All analyses were conducted using R 3.6.1^65^. Package MuMin was used to calculate AIC*c*^62^, and multcomp to perform a Tukey’s post hoc test^66^.

### Animal handling and ethics

The research was conducted in accordance with the guidelines of the Canadian Council on Animal Care. All protocols were reviewed and approved by the University Committee on Animal Care and Supply – Environment and Climate Change Canada's Animal Care Committee, Canada (EC-PN-07-008 (2007), EC-PN-08-026 to EC-PN-12-026 (2008 to 2012), EC-PN-13-0 (2013), EC-PN-14-026 to H.G.G.), the University Committee on Animal Care of the University of Windsor, Canada (AUPP 11-06 to O.P.L.), Animal Research Ethics Board of the University of Saskatchewan, Canada (20100063 to C.S.).

## Supplementary Information

Supplementary Information.

## Data Availability

Annual number of Northern common eiders sampled and annual colony size: uploaded as online Supplementary Table S2. PCR and serology information are available from the Dryad Digital Repository: 10.5061/dryad.tdz08kpzc.
