# Supplementary material for: Herd immunity drives the epidemic fadeout of avian cholera in Arctic-nesting seabirds
Source: Sci Rep. 2021 Jan 13;11:1046. doi: 10.1038/s41598-020-79888-6 (PMC7806777; doi:10.1038/s41598-020-79888-6)
Supplement: Supplementary file 1 — Supplementary Information. [file 41598_2020_79888_MOESM1_ESM.docx]

Supplementary information: Herd immunity drives the epidemic fadeout of avian cholera in Arctic-nesting seabirds

Jacintha G.B. van Dijk^1,2^, Samuel A. Iverson^1,3^, H. Grant Gilchrist^4^, N. Jane Harms^5,6^, Holly L. Hennin^4,7^, Oliver P. Love^7^, E. Isabel Buttler^1^, Stephanie Lesceu^8^, Jeffrey T. Foster^9^, Mark R. Forbes^1^ & Catherine Soos^5,10,*^

^1^Department of Biology, Carleton University, Ottawa, ON K1S 5B6, Canada

^2^Centre for Ecology and Evolution in Microbial Model Systems, Linnaeus University, Kalmar, SE-391 82, Sweden

^3^Environment and Climate Change Canada, Canadian Wildlife Service, Gatineau, QC K1A 0H3 Canada

^4^Environment and Climate Change Canada, National Wildlife Research Center, Ottawa, ON K1S 5B6, Canada
^5^Department of Veterinary Pathology, University of Saskatchewan, Saskatoon, SK S7N 5B4, Canada

^6^Environment Yukon, Animal Health Unit, Whitehorse, YT Y1A 4Y9, Canada

^7^Department of Integrative Biology, University of Windsor, Windsor, ON N9B 3P4, Canada

^8^IDvet, 34790 Grabels, France

^9^Pathogen and Microbiome Institute, Northern Arizona University, Flagstaff, AZ 86011, USA

^10^Environment and Climate Change Canada, Ecotoxicology and Wildlife Health Division, Saskatoon, SK S7N 0X4, Canada

*Correspondence author: catherine.soos@canada.ca

**
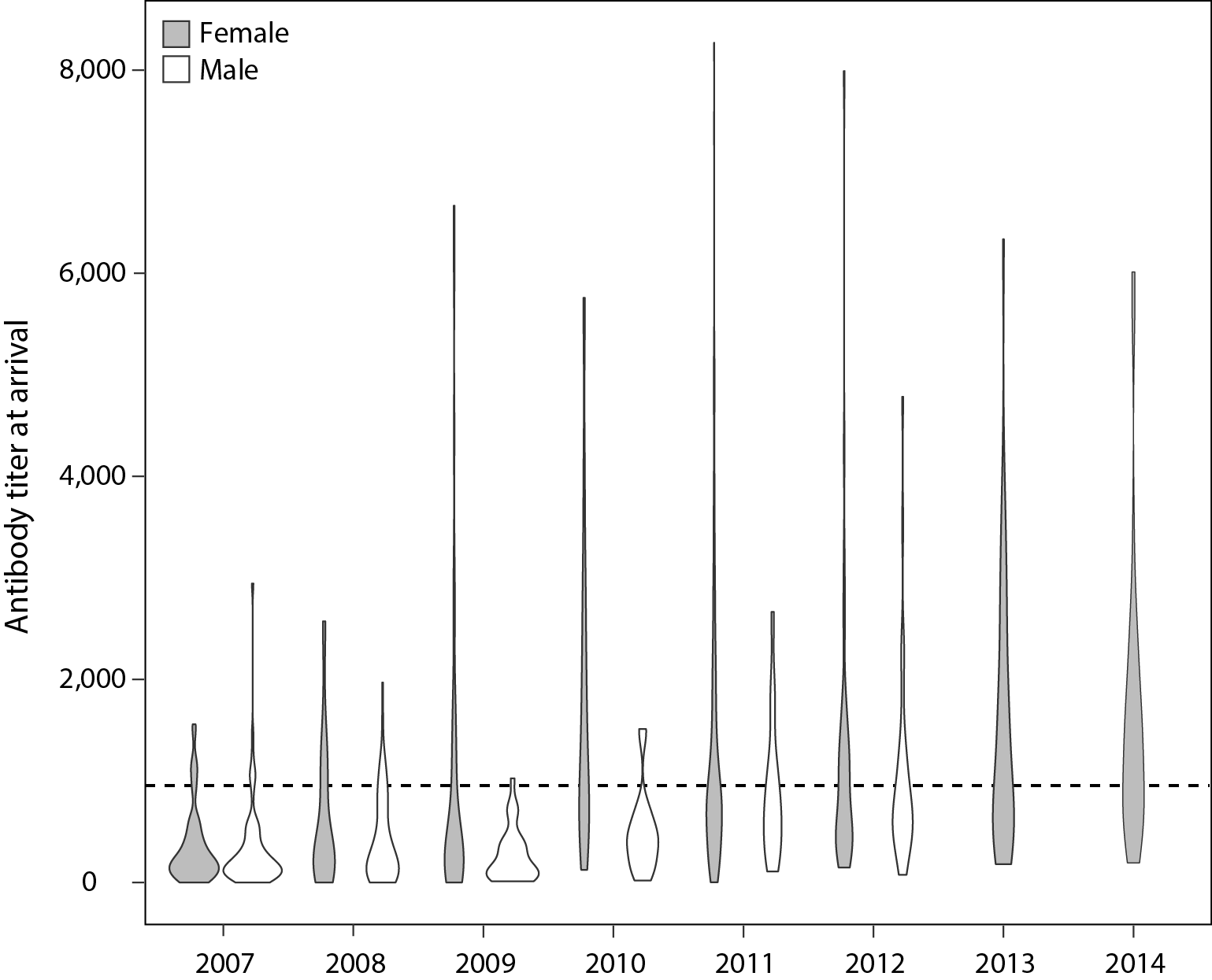
**

**Figure S1.** Raw data of titers of antibodies to *Pasteurella multocida* of apparently healthy Northern common eiders upon arrival to Mitivik Island from 2007-2014, subdivided by year and sex. The dotted line depicts the cut-off point, with titers > 997 considered positive.

**Table S1.** Akaike Information Criterion for small sample sizes (AIC*c*) model selection results. AIC*c* were used to assess the contribution of the explanatory variables measured from 2007–2012 in Northern common eiders at Mitivik Island, Nunavut (i.e., apparent prevalence of *P. multocida* infection upon arrival, seroprevalence upon arrival, antibody titer upon arrival, colony size during the breeding season), in explaining the real-time reproductive number (R_t_) assessed annually for the same period. We identified explanatory variables as non-informative when their ΔAIC*c* were >2 units relative to the null model. Estimates (β) and unconditional SE (incorporates model selection uncertainty) are full averages. K: number of parameters, logLik: log likelihood.

| Explanatory variable | Β | SE | K | logLik | AIC*c* | ∆AIC*c* |
| --- | --- | --- | --- | --- | --- | --- |
| Average annual antibody titer of both sexes | -0.0013 | 0.0006 | 3 | 1.30 | 15.41 | 0.00 |
| Annual seroprevalence of both sexes | -0.028 | 0.022 | 3 | 0.17 | 17.65 | 2.24 |
| Average annual antibody titer of females | -0.0008 | 0.0007 | 3 | 0.09 | 17.82 | 2.41 |
| Null model (intercept) |  |  | 2 | -5.52 | 19.03 | 3.62 |
| Annual seroprevalence of females | -0.016 | 0.021 | 3 | -0.83 | 19.66 | 4.25 |
| Colony size | 0.000 | 0.000 | 3 | -3.88 | 25.76 | 10.35 |
| Average annual antibody titer of males | -0.00004 | 0.0003 | 3 | -4.08 | 26.17 | 10.76 |
| Annual seroprevalence of males | -0.000 | 0.005 | 3 | -4.67 | 27.33 | 11.92 |
| Annual apparent prevalence of  *P. multocida* infection in males | -0.000 | 0.012 | 3 | -5.10 | 28.20 | 12.79 |
| Annual apparent prevalence of  *P. multocida* infection in both sexes | -0.000 | 0.011 | 3 | -5.44 | 28.88 | 13.47 |
| Annual apparent prevalence of  *P. multocida* infection in females | 0.000 | 0.011 | 3 | -5.50 | 29.01 | 13.60 |

**Table S2.** Infection status, serostatus and colony size. Number of samples collected from apparently healthy Northern common eiders captured upon arrival to Mitivik island, Nunavut, used to calculate annual apparent prevalence of *P. multocida* infection, annual seroprevalence, and average annual antibody titer. Annual colony size is defined as the number of common eider breeding pairs.

| Year | Infection status | | Serostatus | | | Colony size |
| --- | --- | --- | --- | --- | --- | --- |
|  | N | Apparent prevalence (%) | N | Seroprevalence (%) | Antibody titer |  |
| 2007 | 472 | 6.1 | 168 | 8.9 | 342 | 5652 |
| 2008 | 430 | 2.8 | 123 | 17.9 | 511 | 5283 |
| 2009 | 475 | 0.6 | 78 | 19.2 | 700 | 3921 |
| 2010 | 420 | 10.0 | 100 | 46.0 | 1467 | 4570 |
| 2011 | 469 | 3.6 | 222 | 39.2 | 1176 | 4545 |
| 2012 | 90 | 0.0 | 87 | 36.8 | 1086 | 4570 |
| 2013 |  |  | 44 | 50.0 | 1499 |  |
| 2014 |  |  | 44 | 61.4 | 1615 |  |

**Table S3.** Pairwise correlation coefficients between explanatory variables.

| Explanatory variables | R | t-value | P-value | r^2^ |
| --- | --- | --- | --- | --- |
| Annual apparent prevalence of *P. multocida* infection *  annual seroprevalence | 0.26 | 0.528 | 0.625 | -0.17 |
| Annual apparent prevalence of *P. multocida* infection *  average annual antibody titer | 0.31 | 0.650 | 0.551 | -0.13 |
| Annual apparent prevalence of *P. multocida* infection *  annual colony size | 0.33 | 0.704 | 0.520 | -0.11 |
| Annual seroprevalence * average annual antibody titer | 0.99 | 13.94 | <0.001 | 0.97 |
| Annual seroprevalence * annual colony size | -0.50 | -1.151 | 0.314 | 0.06 |
| Average annual antibody titer * annual colony size | -0.55 | -1.332 | 0.254 | 0.13 |

**Supplementary Methods: Bacterial detection**

The presence of *P. multocida* was detected in cloacal and oral samples collected from Northern common eiders at Mitivik Island, Nunavut, using a 5’ Taq nuclease PCR assay^1^. DNA was extracted from the 2007–2009 samples using either the Chelex extraction protocol (N=1,250) or a Qiagen kit (N=127) following manufacturer’s guidelines (Qiagen Inc, Valencia, CA, USA). For details on DNA extraction and amplification see Legagneux *et al.*^2^. For the 2010–2012, the Chelex extraction protocol was also used to extract DNA, but with the modification that 10 μL of TSB glycerol from each sample was added to 80 μL of 5% Chelex solution. DNA was amplified as described by Legagneux *et al.*^2^, but with the following modifications: DNA was amplified in 25 μL PCR mixtures containing 2 μL of template DNA, 12.5 μL mastermix (iQ Supermix, Biorad, Mississauga, ON, Canada), 10 pmol of the forward and reverse primers (Sigma-Aldrich, Oakville, ON, Canada), 0.5 μL of TaqMan probe at 10 μM (Applied Biosystems, Life Technologies Inc, Grand Island, NY, USA), and 5.5 μL of ultrapure water. A Biorad iQ5 real-time PCR machine (Applied Biosystems Life Technologies Inc, Grand Island, NY, USA) was used for the 2010–2012 samples. Instead of a single positive control, two positive controls were used on the PCR plates of the 2010–2012 samples: a *P. multocida* isolate from a dead eider from Mitivik Island found in 2008 and 2 μL of *P. multocida* plasmid solution. For the 2007–2009 samples we tested the limits and sensitivity of the assay using serial twelve-fold dilutions, starting at a concentration of 1 ng/uL *P. multocida* DNA and a detection limit of 1x10^-8^ ng/uL^2^. For the 2010–2012 samples, the limits and sensitivity of the assay were tested using serial ten-fold dilutions of the sequence of *P. multocida* DNA in *Escherichia coli* vectors starting at a concentration of 4.23x10^10^ copies of the amplicon/μL, with a detection limit of the assay of 4.23 amplicons/μL. All 2007–2012 samples were classified as *P. multocida* positive if the cycle threshold value was 38 or less^1^.

**References**

1 Corney, B. G. *et al.* *Pasteurella multocida* detection by 5′ Taq nuclease assay: a new tool for use in diagnosing fowl cholera. *J. Microbiol. Methods* **69**, 376-380, <https://doi.org/10.1016/j.mimet.2007.01.014> (2007).

2 Legagneux, P. *et al.* No selection on immunological markers in response to a highly virulent pathogen in an Arctic breeding bird. *Evol. Appl.* **7**, 765-773, <https://doi.org/10.1111/eva.12180> (2014).
